# Evaluating Stroke-Related Motor Impairment and Recovery Using Macroscopic and Microscopic Features of HD-sEMG

**DOI:** 10.3390/bioengineering12121357

**Published:** 2025-12-12

**Authors:** Wenting Qin, Xin Tan, Yi Yu, Yujie Zhang, Zhanhui Lin, Chenyun Dai, Yuxiang Yang, Lingyu Liu, Lingjing Jin

**Affiliations:** 1 The Department of Neurology and Neurological Rehabilitation, Shanghai Disabled Persons’ Federation Key Laboratory of Intelligent Rehabilitation Assistive Devices and Technologies, Yangzhi Rehabilitation Hospital (Shanghai Sunshine Rehabilitation Center), School of Medicine, Tongji University, Shanghai 200125, China; 2280271@tongji.edu.cn; 2 The College of Engineering and Design, Hunan Normal University, Changsha 410081, China; 3 The School of Information Science and Technology, Fudan University, Shanghai 200433, China; 4 The School of Biomedical Engineering, Shanghai Jiao Tong University, Shanghai 200241, China

**Keywords:** stroke, tremor, high-density surface electromyography, motor unit

## Abstract

Stroke-induced motor impairment necessitates objective and quantitative assessment tools for rehabilitation planning. In this study, a gesture-specific framework based on high-density surface electromyography (HD-sEMG) was developed to characterize neuromuscular dysfunction using eight macroscopic features and two microscopic motor unit decomposition features. HD-sEMG recordings were collected from stroke patients (*n* = 11; affected and unaffected sides) and healthy controls (*n* = 8; dominant side) during seven standardized hand gestures. Feature-level comparisons revealed hierarchical abnormalities, with the affected side showing significantly reduced activation/coordination relative to healthy controls, while the unaffected side exhibited intermediate deviations. For each gesture, dedicated K-nearest neighbors (KNN) models were constructed for clinical validation. For Brunnstrom stage classification, wrist extension yielded the best performance, achieving 92.08% accuracy and effectively discriminating severe (Stage 4), moderate (Stage 5), and mild (Stage 6) impairment as well as healthy controls. For fine motor recovery prediction, the thumb–index–middle finger pinch provided the optimal regression performance, predicting Upper Extremity Fugl–Meyer Assessment (UE-FMA) scores with R = 0.86 and RMSE = 3.24. These results indicate that gesture selection should be aligned with the clinical endpoint: wrist extension is most informative for gross recovery staging, whereas pinch gestures better capture fine motor control. Overall, the proposed HD-sEMG framework provides an objective approach for monitoring post-stroke recovery and supporting personalized rehabilitation assessment.

## 1. Introduction

Stroke is a common neurological disease [1,2] and remains a significant global health burden. In 2019, globally, it was the second-leading cause of death, accounting for 11.6% of total deaths, and the third-leading cause of death and disability combined, contributing to 5.7% of total disability-adjusted life-years [3,4]. One of the major sequelae of stroke is upper limb motor impairment, with hand function being particularly affected [5]. Therefore, a precise evaluation of the hand motor impairments is crucial for designing effective rehabilitation strategies and treatment plans to enhance the quality of life for stroke survivors.

Currently, assessment of hand motor function in stroke patients primarily relies on standardized clinical scales, such as the Brunnstrom stages and the Fugl-Meyer Assessment (FMA). While these tools are widely used, they are inherently subjective, intermittent, and influenced by clinician variability and patient factors, which limits their accuracy and real-world applicability. High-density surface electromyography (HD-sEMG) has emerged as a promising tool to complement traditional assessments, offering non-invasive, multi-channel, high-resolution recordings of neuromuscular activity [6,7]. Compared with intramuscular EMG or conventional surface EMG, HD-sEMG enables detailed mapping of motor unit (MU) activity and coordination patterns that are difficult to capture with clinical scales alone. This capability provides a quantitative, objective basis for understanding stroke-induced motor dysfunctions and guiding personalized rehabilitation.

Recent studies have increasingly highlighted the potential of HD-sEMG for understanding stroke-related neuromuscular changes in the spatial and temporal distribution of muscle activity. For example, Xie et al. compared the spatial distribution and intensity of muscle activation in spastic hemiparetic individuals compared to healthy controls, revealing velocity-dependent heterogeneous activation during passive stretch and active contraction [8]. Similarly, Zhang et al. analyzed temporal and spatial variations of neuromuscular activities in stroke patients, identifying altered bilateral synergy and coordination in those with upper limb motor impairment [9]. Beyond general neuromuscular activity, the investigation of MUs distribution and recruitment patterns in muscle activation is another key factor in understanding neuromuscular function and stroke-induced dysfunctions. Xie et al. explored the temporal patterns and spatial heterogeneity of MUs recruitment in spastic muscles during passive stretch, revealing reduced temporal variability and activation patterns correlated with spasticity severity [10]. Ruan et al. systematically quantified MUs’ action potential distributions and co-activation patterns, showing increased co-activation in extrinsic muscles and altered MU distributions linked to impaired finger independence [11]. Liu et al. further advanced this field by characterizing MU distribution and recruitment patterns in the spastic and non-spastic biceps brachii muscles of chronic stroke survivors, demonstrating disrupted force-related recruitment and inter-side differences in MU depth [7].

Despite these advances, existing HD-sEMG-based assessment approaches face several limitations. First, systematic comparisons of HD-sEMG features between affected and unaffected sides of stroke patients, as well as with healthy controls, remain limited. Second, most studies focus either on macroscopic or microscopic features, without integrating both levels to capture the full spectrum of neuromuscular activity. Third, the clinical relevance of extracted HD-sEMG features has not been consistently validated against established scales, restricting their applicability in rehabilitation practice. Lastly, gesture-specific analyses are scarce, leaving task-specific neuromuscular dysfunctions—critical for fine motor rehabilitation—poorly understood. Addressing these gaps is essential for translating HD-sEMG findings into clinically actionable insights.

To address the challenges in accurately assessing stroke-induced motor impairment, this study evaluates eight macroscopic and two microscopic features to quantify hand function and the degree of hand impairment in stroke survivors. First, we explore stroke-related neuromuscular changes by analyzing feature distributions across the affected and unaffected sides of stroke patients, as well as the dominant side of healthy controls. This analysis reveals distinct differences in muscle coordination and strength between these groups. Second, using the KNN approach based on these features, we developed a classification model for Brunnstrom stages and a regression model for predicting UE-FMA scores, demonstrating the validity of these features in clinical assessments of motor impairment. Third, we evaluate specific gestures that are highly predictive of Brunnstrom stages and UE-FMA scores in stroke patients, and remain robust in distinguishing healthy neuromuscular patterns in controls. The main innovations of this work are as follows:

(1) Comprehensive Feature Analysis: We provide a holistic perspective by integrating both macroscopic and microscopic features to analyze neuromuscular signals, revealing significant differences between stroke patients and healthy controls that reflect the underlying physiological mechanisms.
(2) Feature-Based Robust Modeling: Using these features, we developed a KNN-based classification and regression model that accurately classifies Brunnstrom stages and predicts UE-FMA scores, demonstrating their robustness and clinical applicability for assessing motor impairment.
(3) Gesture-Specific Insights: By identifying gestures that are highly predictive of recovery stages and UE-FMA scores, we highlight the task-specific nature of neuromuscular dysfunctions, offering precise insights for personalized rehabilitation planning.

## 2. Materials

### 2.1. Subjects

Nineteen subjects participated in this study, including 11 stroke patients with varying degrees of bradykinesia (ten males and 1 female; aged 27–75 years with a mean age 54.27 ± 16.01 years) and 8 healthy subjects (five males and three females; aged 30–66 years with a mean age of 53.88 ± 10.84 years) with no symptoms of hand movement disorders. All stroke patients, as assessed by a neurologist, exhibited marked difficulty in performing hand movements with their single affected limb. Detailed clinical information about the stroke patients, including gender, age, stroke duration, affected side, etiology, Brunnstrom stage, and UE-FMA, is shown in Appendix A. The Brunnstrom stage indicates motor recovery, with higher stages reflecting better recovery and advanced voluntary movement control following a stroke [12]. The UE-FMA is a subset of the full FMA, specifically assessing the distal upper limb (shoulder, elbow, and fingers). It includes 15 items, each scored on a 3-point scale (0 = cannot perform, 1 = performs partially, 2 = performs fully), with a maximum score of 30. Higher scores signify better motor function recovery [12]. All participants provided informed consent before the study, which was approved by the Ethics Committee of Shanghai Yangzhi Rehabilitation Hospital (approval number: YZ 2024-140).

### 2.2. Data Acquisition

**Figure 1:**
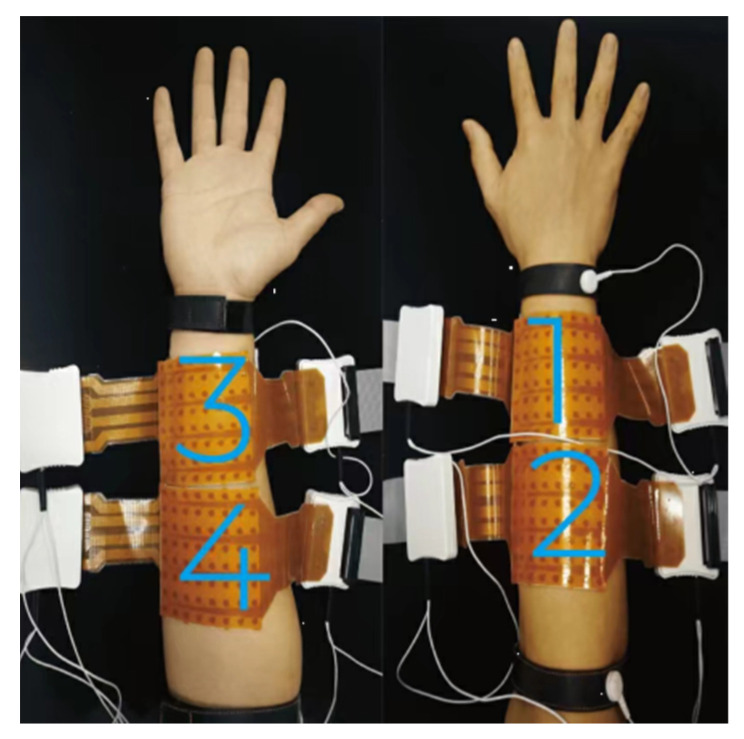
Electrode placement. Each brown ellipse indicates a single sEMG channel. This figure is adapted from our open-access paper [13], available under the Creative Commons License (https://creativecommons.org/licenses/by/4.0/).

The HD-sEMG signals were recorded using the Quattro-cento system (OT Bioelettronica, Turin, Italy) at a sampling rate of 2048 Hz, a gain of 150, and a resolution of 16 bits. To minimize skin-electrode impedance, the forearm of the recording side was prepped with abrasive gel and cleaned with water before data collection. Four 8 × 8 HD-electrode arrays (Adhesive Matrix ELSCH064NM1, OT Bioelettronica, Turin, Italy) were placed on the forearm, as illustrated in Figure 1. Each electrode array featured a 10-mm inter-electrode distance with elliptical electrode points (major axis: 5 mm, minor axis: 2.8 mm). The 256 recording channels were created by concatenating the 64 channels from arrays 1, 2, 3, and 4, as labeled in Figure 1. Specifically, the radial and ulnar sides of the forearm, the humeroulnar joint, and the head of the ulna serve as the boundaries of the area where the electrode array is placed. On each forearm side, two adjacent electrode arrays were combined to form a 16 × 8 electrode configuration, with the adjoining sides of the arrays touching. Arrays 1 and 2 covered the extensor muscles, and arrays 3 and 4 covered the flexor muscles. The long axis of the 16 × 8 arrays was aligned with the long axis of the forearm, ensuring the center of the electrode placement matched the center of the array configuration. The right leg drive electrode was positioned at the head of the ulna, and the reference electrode was placed on the olecranon.

### 2.3. Experimental Paradigm

**Figure 2:**
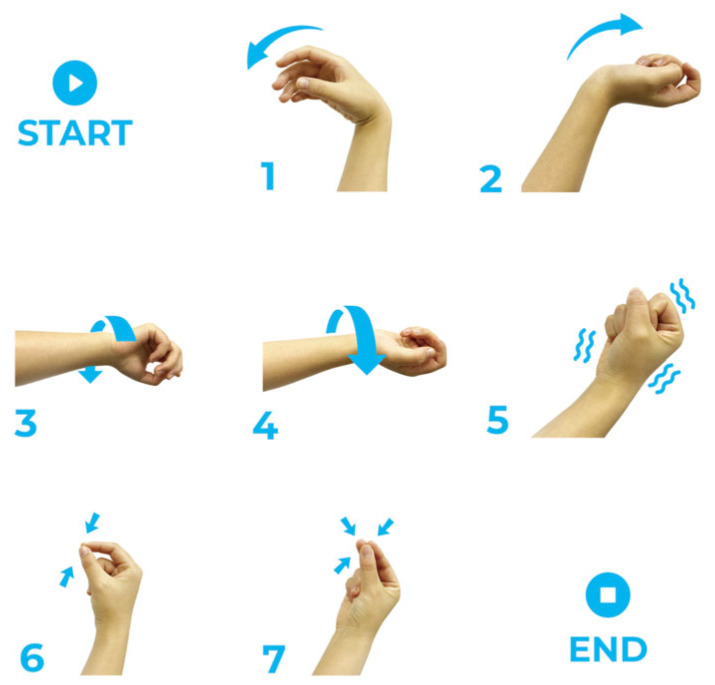
All involved gestures: (1) Wrist Flexion (WF), (2) Wrist Extension (WE), (3) Wrist Pronation (WP), (4) Wrist Supination (WS), (5) Hand Close (HC), (6) Thumb and Index Fingers Pinch (TIFP), (7) Thumb, Index and Middle Fingers Pinch (TIMFP).

During data acquisition, participants sat comfortably and followed the instructions displayed on a computer screen to perform the seven hand gestures shown in Figure 2. Specifically, wrist flexion (WF) primarily engages the flexor carpi radialis and flexor carpi ulnaris muscles, and wrist extension (WE) involves the extensor carpi radialis and extensor carpi ulnaris muscles, which are crucial for grasping and releasing objects. Wrist Pronation (WP) engages the pronator muscles, and wrist Supination (WS) involves the supinator muscle. Hand Close (HC) utilizes the flexor digitorum muscles, which are a fundamental component of grip strength and dexterity. Thumb and index finger pinch (TIFP) primarily involves the flexor pollicis muscles and the first dorsal interosseous. The TIFP is critical for precision tasks, which assess fine motor control and coordination. Thumb, index, and middle fingers pinch (TIMFP) engage the flexor pollicis muscles, flexor digitorum superficialis, and the intrinsic hand muscles. This TIMFP extends the assessment of pinch strength and dexterity, important for slightly larger objects and more complex manipulation tasks. These seven gestures correspond to movements that are vital for daily activities and cover a wide range of motor functions that are commonly impaired in stroke patients. Each gesture involves different muscle groups, allowing for a comprehensive evaluation of muscle strength and control in the wrist and hand. Before the experiment, the subjects could practice switching between different hand gestures until they could complete each gesture task as accurately as possible. The hand gesture instructions were randomized and presented one at a time. Each subject performed two repeated blocks for each gesture. In each block, subjects performed 3 dynamic task trials and 1 maintenance task trial. Dynamic tasks involved transitioning from a resting state to the required gesture state.

Maintenance tasks involved transitioning from a resting state to the required gesture, followed by maintenance of the gesture. Due to variations in the abilities of subjects, the duration of each data segment varied dynamically. Generally, dynamic tasks and maintenance tasks had to last at least 1 s and 3 s, respectively. Subjects were given a 2-s rest period between trials and a 5 s rest period between blocks to avoid muscle fatigue. An audible beep indicated the start of each trial. At the onset of each trial, a trigger signal was sent synchronously, which could be used for sEMG segmentation. For each stroke subject, HD-sEMG signals were acquired during 84 dynamic trials (2 sides × 7 hand gestures × 2 blocks × 3 trials) and 28 maintenance trials (2 sides × 7 hand gestures × 2 blocks × 1 trial). For each healthy subject, HD-sEMG signals were acquired during 42 dynamic trials (7 hand gestures × 2 blocks × 3 trials) and 14 maintenance trials (7 hand gestures × 2 blocks × 1 trial). If subjects missed or performed a trial incorrectly, they informed the experiment assistant, who could use the redo button on the guide instructions for the subject to re-perform the skipped or incorrect trials. For healthy subjects, only data acquisition was conducted on the dominant side (all healthy subjects were right-handed). For stroke patients, data acquisition was performed on both the unaffected and affected sides on the same day, adhering to the same experimental protocol.

## 3. Method and Analysis

### 3.1. Data Processing

The HD-sEMG signals were preprocessed using an eighth-order zero-phase Butterworth band-pass filter implemented in the MATLAB Signal Processing Toolbox (Version R2023b; MathWorks, Natick, MA, USA). The filter was applied with a high-pass cutoff frequency of 10 Hz and a low-pass cutoff frequency of 500 Hz, a configuration widely adopted in sEMG research [13,14]. Zero-phase filtering was achieved by processing the signals forward and backward to eliminate phase distortion. In addition, notch filters were applied at 50 Hz and its harmonic frequencies to remove power line interference while preserving signal integrity within the 10–500 Hz bandwidth [15].

### 3.2. Feature Definitions

We categorized the extracted features into two groups based on their physiological information source and complexity: Macroscopic Features and Microscopic Features.

Macroscopic Features are derived from the aggregate analysis of multi-channel raw or smoothed sEMG signals. They reflect the overall activity of the entire muscle, representing gross muscle effort, power, and coordination—the collective, observable electrical output.

Microscopic Features are derived from Motor Unit Decomposition or complex non-linear analysis. They are intended to capture the underlying neural control strategies and complexity at the motor unit level. For instance, complexity metrics reflect the non-linear, temporal structure of motor unit recruitment and firing patterns orchestrated by the central nervous system.

### 3.3. Macroscopic Feature Extraction

The HD-sEMG data acquisition utilized a total of 256 channels arranged in four 8 × 8 electrode arrays. Feature extraction was performed on a channel-by-channel basis; therefore, no spatial mapping or interpolation strategies were used for the 2D sEMG analysis. Furthermore, all macroscopic features listed below were computed over the entire duration of the sustained contraction segment for each hand gesture, without using small time-domain windows for feature extraction.

This study extracted eight macroscopic features from preprocessed HD-sEMG signals to assess muscle function during stroke rehabilitation. These features capture key physiological aspects such as muscle strength, energy distribution, activity patterns, and coordination, which are essential for evaluating muscle functionality and dysfunction.

(1) Complexity and Variability: These features capture muscle contraction dynamics, crucial for understanding responses to movement and rehabilitation.
  - Average Amplitude Change (AAC) [16]: Measures the average change in signal magnitude between consecutive data points, reflecting muscle response variability during rehabilitation.
  - Differential Average Standard Deviation Variability (DASDV) [17]: Quantifies the temporal variability of muscle activation by computing the standard deviation of the rectified (absolute) EMG amplitude across consecutive samples. This metric reflects short-term fluctuations in the signal envelope, which correspond to changes in muscle activity over time.
(2) Muscle Strength: These features were selected to measure the overall muscle activation levels.
  - Mean Absolute Value (MAV) [18]: Represents the average of the rectified EMG amplitude, providing a surrogate index of overall muscle activation.
  - Average Spectral Magnitude (ASM) [19]: Quantifies the mean amplitude of the power spectrum of the sEMG signal, computed using Welch’s averaged periodogram method (Hamming window, 250 ms segment length, 50% overlap). ASM provides a compact representation of the overall spectral power distribution and is often used to characterize the general frequency composition of muscle activity.
    Root Mean Square (RMS) [14]: Represents the square root of the mean power of the sEMG signal, reflecting the overall level of muscle activation.
(3) Energy Distribution and Amplitude Variations: These features evaluate how muscle contraction energy varies across time and different segments of the signal.
  - Modified Hamming Window (MHW) [20]: By applying a Hamming window function, this feature captures the temporal changes in signal energy. It helps assess how the muscle distributes energy during contractions and understand muscle function during repetitive or sustained activities.
  - Modified Trapezoidal Window (MTW) [21]: Similar to the MHW but uses a trapezoidal window. It is designed to provide additional insights into how muscle contractions vary in amplitude over time, complementing the energy distribution captured by the MHW.
(4) Coordination and Synchronization: This feature was selected to evaluate the muscle coordination between different muscle regions.
  - Synchronization (Sync): Measures the correlation between signals from neighboring channels, indicating how well different regions of the muscle coordinate their activity.

### 3.4. Microscopic Feature Extraction

**Figure 3:**
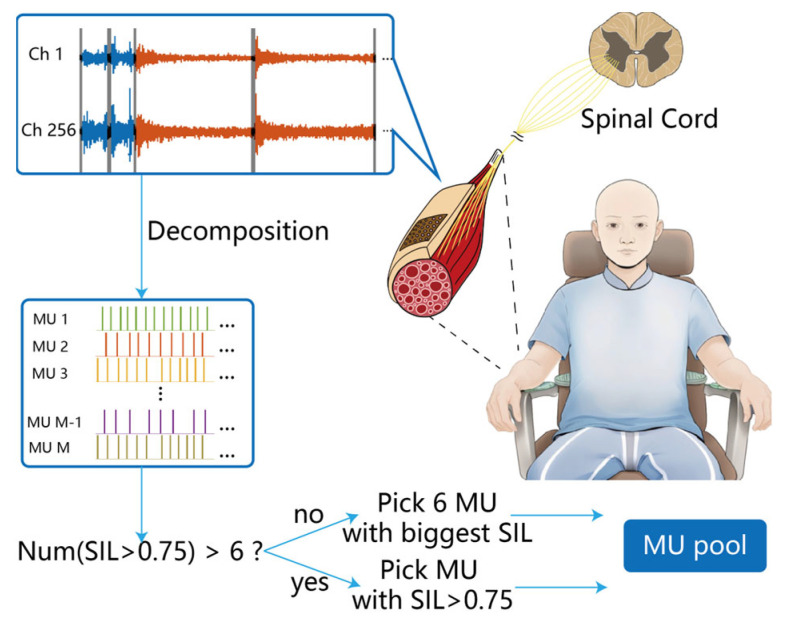
Block diagram of the MU decomposition and selection.

Microscopic features were extracted from the preprocessed HD-sEMG signals by decomposing the signals into individual MU spike trains using the fast independent component analysis (FastICA) algorithm [22,23]. The diagram of MU decomposition and selection is shown in Figure 3. It includes three main steps:

(1) Concatenation of HD-sEMG data. The HD-sEMG data is concatenated to facilitate more effective MU decomposition. The FastICA algorithm, which relies on the central limit theorem and maximization of non-Gaussianity, requires a sufficient length of data for accurate independent component extraction [24]. Since the same gesture typically exhibits consistent muscle activation patterns across repeated trials, we concatenate samples of the same gesture (6 dynamic trials and 2 maintenance trials) to enhance MU decomposition. To ensure smooth transitions between concatenated data segments, 100 sample points from the resting state (shown as the black signal segment in Figure 3) are extracted before and after each sample during concatenation. In Figure 3, the dynamic trials are represented by the blue signal segments in the upper-left corner, while the maintenance trials are shown as the orange signal segments.

(2) MU Decomposition. Extend the registration HD-sEMG signals by adding delayed replicas of each original channel. Whiten the extended signals with the eigenvalue decomposition, which ensures that the processed data have zero mean and unit variance. Deconvolute the whitened signals using the FastICA algorithm [25,26].
(3) MU detection and selection. The discharge timings of MUs are detected using k-means clustering, and the clustering index silhouette (SIL) is calculated to evaluate the decomposition performance. The SIL measures the distinctness of the extracted MU in comparison to other MUs and background noise during clustering. A higher SIL value indicates better separation from the baseline, and it is calculated as:
  (1) $$SIL=\frac{1}{N}\sum_{i=1}^{N}\frac{b\left(i\right)-a(i)}{max(a\left(i\right),b(i))}$$
  where a(i) is the average distance from the i-th point to the other points in the same cluster, and b(i) is the average distance from the i-th point to points in the nearest cluster. Following the SIL calculation, duplicate MUs are removed. The FastICA algorithm may detect both the original MU and its delayed replicas. If a pair of spike trains shows 50% synchronized discharge within ±1 ms after time alignment, only the MU spike train with the higher SIL value is retained for further analysis [27]. For MU selection, if the number of MUs with an SIL greater than 0.75 [28] is fewer than 6, the top 6 MUs with the highest SIL values are included in the MU pool. If more than 6 MUs have an SIL greater than 0.75, all MUs with an SIL greater than 0.75 are included in the MU pool. A rigorous manual calibration was performed to check sources and spike trains exceeding the SIL threshold, ensuring stability and consistency in inter-spike intervals and amplitudes. Physiological discharge patterns were confirmed by verifying inter-spike intervals between 20 ms (reflecting the refractory period of human motor neurons) and 250 ms [23]. Sparseness, inversely related to inter-spike intervals, was also assessed to meet the assumptions of blind source separation algorithms. Only spike trains with stable morphology, amplitude, and physiological intervals were included for further analysis.

Two key microscopic features were extracted from the MU decomposition process.

(1) The Number of MUs (NMUs): NMUs denotes the total number of detected and retained motor units per gesture after the above selection procedure. Across gestures in our dataset, the average NMUs decomposed per gesture typically ranged from 30 to 40. Moreover, the approximate proportion of detected MUs with SIL > 0.75 was around 25%, 10%, and 6% for the affected side, unaffected side, and healthy controls, respectively. The NMUs can reflect the level of muscle recruitment and are crucial for understanding muscle function and control.
(2) Average Firing Rate (AFR): The AFR is the mean of the instantaneous firing rate. The instantaneous firing rate can be calculated by:
  (2) $$AFR=\frac{1}{∆n/fs}$$
  where ∆n represents the number of sampling points between two consecutive discharge moments, and f s represents a sampling rate of 2048. AFR provides insights into the neural drive of the muscle. A higher firing rate can indicate greater neural activation and muscle contraction.

### 3.5. Validation Protocol

In this study, three validation protocols were designed to evaluate the performance of the extracted macroscopic and microscopic features in differentiating the affected and unaffected sides in stroke patients, as well as comparing them to the dominant side of healthy controls. In addition, these protocols assessed the effectiveness of these features in predicting motor recovery stages and fine motor control restoration.

The data analysis employed two comparison structures: between-subject comparison was used to contrast the stroke group with the healthy control group; and a within-subject comparison was conducted to analyze the differences between the affected and unaffected sides of the stroke patients.

(1) Protocol 1: This protocol focused on analyzing the distribution of eight macroscopic and two microscopic features across seven distinct hand gestures. The primary goal was to compare muscle activity patterns between three groups: the dominant side of healthy controls, the affected side of stroke patients, and the unaffected side of stroke patients. By examining the features across these groups, we aimed to identify distinct neuromuscular differences related to stroke-induced motor impairment.
(2) Protocol 2: This protocol aimed to evaluate whether the extracted macroscopic and microscopic sEMG features can be used to classify motor recovery stages in stroke patients, using the Brunnstrom stages as clinical reference labels. Whereas we acknowledge that Brunnstrom staging involves a degree of subjectivity, it remains one of the most widely adopted clinical frameworks for assessing upper limb recovery. Data from the affected sides of 11 stroke patients and the dominant sides of 8 healthy controls were collected and aggregated. For each hand gesture, trials were labeled based on the Brunnstrom stages (Stage 4, Stage 5, and Stage 6) or categorized as “healthy” for the healthy controls. The classification accuracy for each gesture was then analyzed to identify which gestures were most informative and sensitive in differentiating between the stages of motor recovery in stroke patients. This analysis aimed to pinpoint gestures that could serve as reliable indicators of motor recovery progress based on the Brunnstrom stages.
(3) Protocol 3: This protocol investigated the potential of the extracted features to predict Upper Extremity Fugl-Meyer Assessment (UE-FMA) scores, a widely accepted clinical metric for quantifying fine motor control in stroke rehabilitation. Specifically, data from each gesture performed by the affected sides of stroke patients were aggregated, with each trial labeled with the corresponding UE-FMA scores. Additionally, data from the dominant sides of healthy controls were included, with these trials assigned a perfect UE-FMA score of 30. The regression analysis focused on calculating the R-value and RMSE for each gesture, identifying key gestures that most accurately predicted UE-FMA scores. The analysis aimed to identify the most predictive gestures for assessing fine motor control recovery.

We utilized the clinically recognized UE-FMA and the Brunnstrom stages as clinical gold standards for assessing motor impairment and functional recovery. Specifically, this study’s Protocol 2 aims to classify Brunnstrom stages using HD-sEMG features, while Protocol 3 aims to predict the continuous UE-FMA score, thereby directly linking the extracted HD-sEMG biomarkers to clinical severity.

### 3.6. Training and Testing

We used a K-nearest neighbors (KNN) algorithm [29] with 10 normalized features for classification in Protocol 2 and regression in Protocol 3. The number of neighbors was set to K = 5 using Euclidean distance. All features were standardized (zero mean, unit variance). To mitigate overfitting and ensure robustness, a leave-one-trial-out cross-validation strategy was adopted. For each gesture, one trial per subject was used as the test set, while the remaining trials from stroke patients and healthy controls were aggregated as training data. This ensures that the model is tested on unseen trials, allowing for generalizable performance assessment.

### 3.7. Statistical Analysis

To quantify differences in the features across the three groups in protocol 1, the Kruskal-Wallis Test [30], a non-parametric method, was used, as the data did not follow a Gaussian distribution. Bonferroni-Holm correction was applied to control for multiple comparisons [31]. Significant differences were reported for *p* < 0.05, using the Bonferroni-Holm corrected *p*-values. All statistical analyses were performed in Python 3.9 using SciPy (v1.15).

## 4. Results

### 4.1. Protocol 1: Macroscopic & Microscopic Feature Analysis

**Figure 4:**
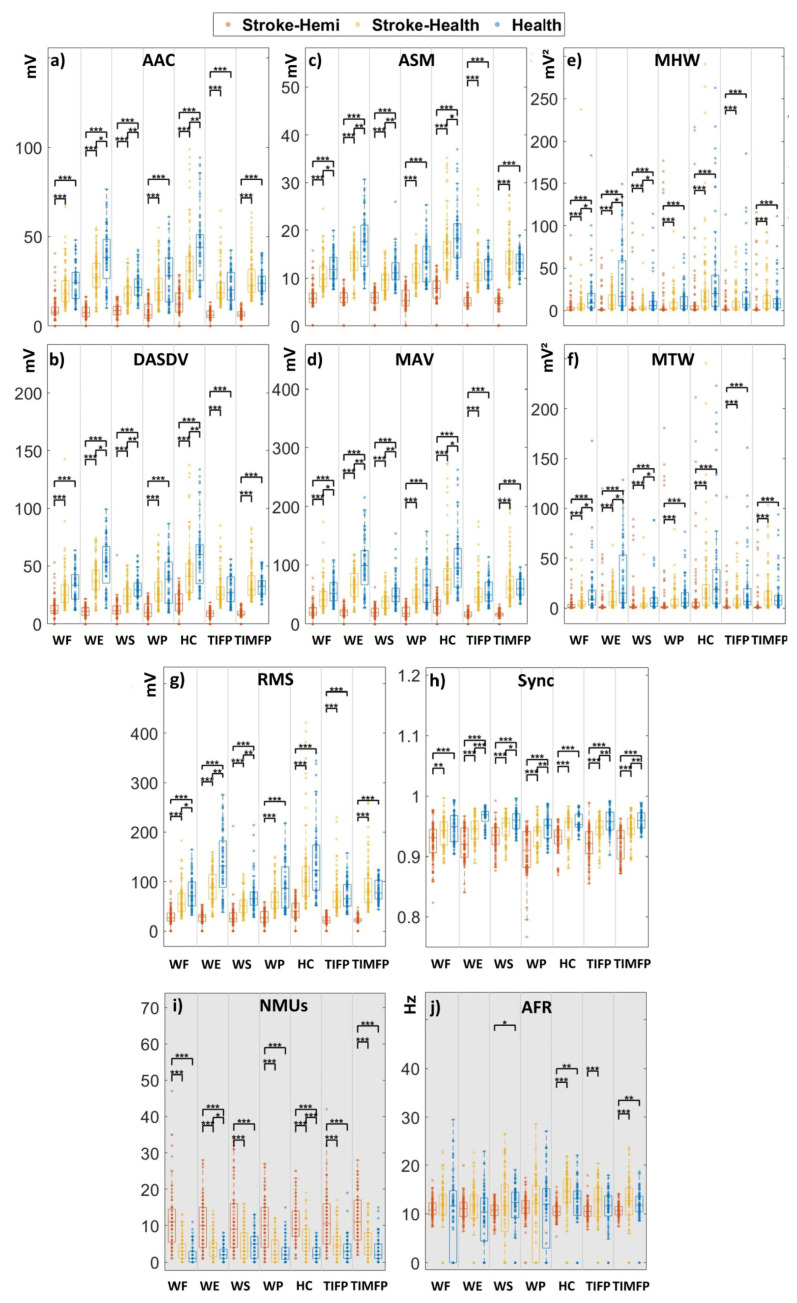
The boxplots of macroscopic and microscopic features across seven gestures for three groups. The boxplots show the distribution of macroscopic and microscopic features across seven gestures for three groups: the dominant side of healthy subjects, the affected side of stroke subjects, and the unaffected side of stroke subjects. Panels (**a**–**h**) show eight macroscopic features, and panels (**i**,**j**) display two microscopic features. Significance levels are indicated as follows: * *p* < 0.05, ** *p* < 0.01, *** *p* < 0.001. The hypothesis suggests significant unilateral dominance in feature values, with macroscopic features and the microscopic AFR feature following the trend: healthy > affected side > unaffected side. In contrast, the NMUs’ microscopic feature follows the opposite trend: healthy < affected side < unaffected side.

The boxplots of eight macroscopic features (AAC, DASDV, MAV, ASM, MHW, MTW, RMS, and Sync) across groups (dominant side of healthy subjects, affected side of stroke subjects, and unaffected side of stroke subjects) in seven gestures are shown in Figure 4a–h. Across all features, the dominant side of healthy subjects shows significantly higher values than the affected sides of stroke subjects (*p* < 0.001). The unaffected sides of stroke subjects also exhibit significantly higher values than the affected sides (*p* < 0.001), except for the Sync feature under the WF gesture (*p* < 0.01). For most macroscopic features, the dominant side of healthy subjects has significantly higher values than the unaffected sides of stroke subjects in about half of the gestures (*p* < 0.001), whereas showing comparable or lower values in the other half. The boxplots of two microscopic features (NMUs and AFR) are shown in Figure 4i,j. The NMUs feature is significantly higher on the affected sides of stroke subjects compared to both the unaffected sides and the dominant sides of healthy subjects (*p* < 0.001). The unaffected sides of stroke subjects show comparable or slightly higher values than healthy subjects. For the AFR feature, the dominant side of healthy subjects exhibits significantly higher values than the affected sides of stroke subjects in about half of the gestures (*p* < 0.05) but comparable values in the remaining gestures. The dominant side of healthy subjects shows values comparable to the unaffected sides of stroke subjects. These general findings indicate that stroke-related changes in motor control are reflected in the sEMG features, with the affected side showing obvious altered muscle activity patterns compared to the unaffected and healthy subjects. These differences highlight the physiological impact of stroke on neuromuscular coordination and the feasibility of considering both macroscopic and microscopic features for evaluating motor impairment in stroke patients. Collectively, Figure 4 serves as the primary feature-level summary of the discriminative patterns across groups for all gestures. These feature-level observations directly motivate and guide the subsequent clinical validation tasks.

### 4.2. Protocol 2: Classification for Brunnstrom Stages

**Figure 5:**
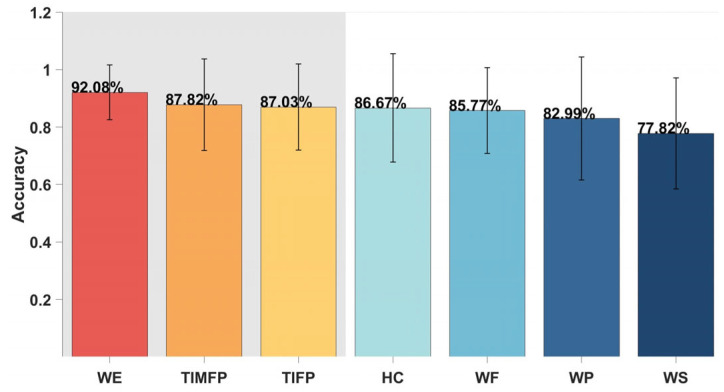
Classification accuracy under different gestures. The error bars represent the standard deviation of the data.

**Figure 6:**
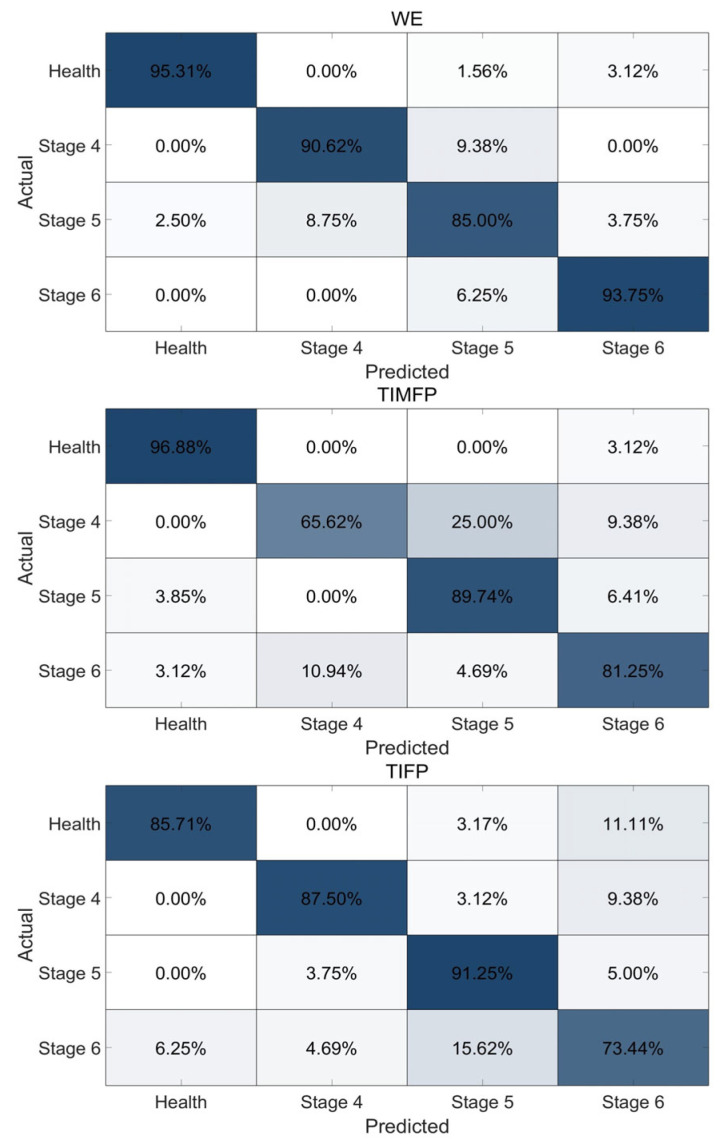
Confusion matrix for the best-performing three gestures.

The top three gestures, WE, TIMFP, and TIFP, achieved high classification accuracies of 92.08%, 87.82%, and 87.0%, respectively, making them the most effective in distinguishing between the Brunnstrom stages of motor recovery in stroke patients and the healthy controls. Other gestures, such as HC (86.67%), WF (85.77%), WP (82.99%), and WS (77.82%), demonstrated slightly lower but still strong accuracies, all above 77%, as shown in Figure 5. The confusion matrix for the HC gesture revealed that misclassifications were most likely to occur between adjacent stages, particularly between Brunnstrom stage 6 and the healthy controls, indicating some overlap in motor abilities at these stages, as shown in Figure 6.

### 4.3. Protocol 3: Regression for UE-FMA Scores

**Figure 7:**
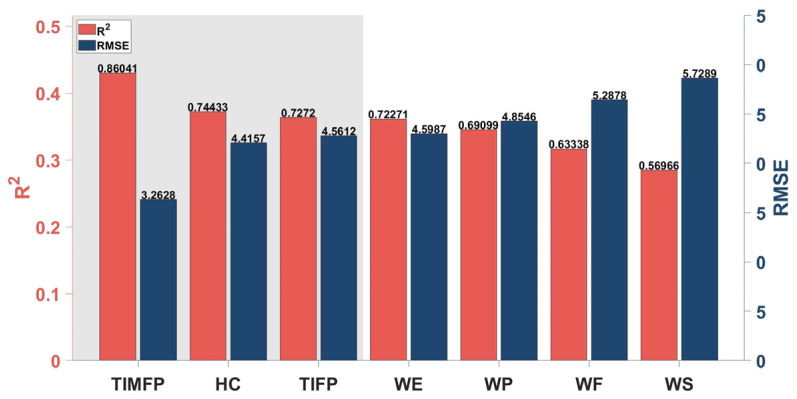
R and RMSE under different gestures.

**Figure 8:**
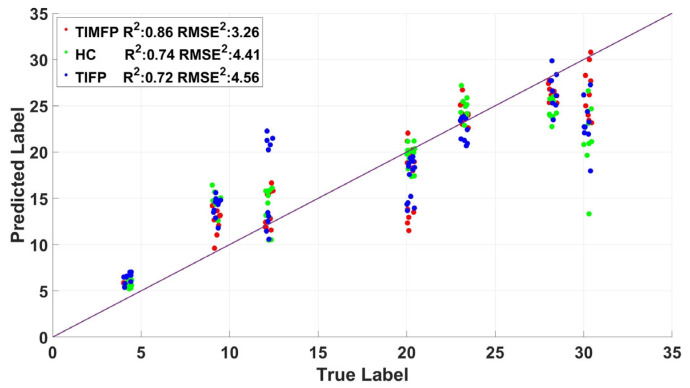
Scatter and residual for the best-performing three gestures.

The R-value and RMSE of the regression model for different gestures are shown in Figure 7. The results showed that the top three gestures, TIMFP, HC, and TIFP, achieved strong R-value values of 0.86, 0.74, and 0.73, respectively, indicating a high correlation with UE-FMA scores, while their RMSE values (3.26, 4.42, and 4.56) indicated relatively low prediction errors. In contrast, gestures like WE, WP, WF, and WS had lower R-value values (ranging from 0.60 to 0.72) and higher RMSE values (ranging from 4.60 to 5.73), suggesting that these gestures were less effective in predicting UE-FMA scores. The scatter, residual, and normal probability plots for the top three gestures are shown in Figure 8, which further support the robustness of TIMFP, HC, and TIFP in accurately predicting recovery. These findings highlight the importance of selecting specific gestures for effective assessment of motor control recovery in stroke patients.

## 5. Discussion

This study aimed to explore the neurophysiological differences between the affected and unaffected sides of stroke patients by extracting macroscopic and microscopic features from HD-sEMG and comparing these differences with healthy controls. We evaluated the effectiveness of these features in both classification and regression tasks, focusing on the classification of motor recovery stages (Brunnstrom stages) and the prediction of fine motor control restoration (UE-FMA scores). The analysis identified key gestures for assessing motor recovery, providing insights into stroke rehabilitation.

The analysis of eight macroscopic features and two microscopic features revealed significant differences in muscle activity patterns between the affected side, unaffected side, and dominant side of healthy subjects. Healthy individuals and the unaffected side of stroke patients exhibit higher values across most macroscopic features and gestures, indicating finely tuned muscle control, greater strength, and better coordination [32]. In contrast, the affected side of stroke patients shows lower macroscopic-feature values, reflecting diminished muscle activity, strength, and coordination [33], which is consistent with previous studies that report reduced muscle strength and impaired motor control in stroke-affected limbs [34]. Specifically, lower macroscopic feature values on the affected side, when performing the same gesture, include: AAC and DASDV, which reflect reduced complexity and variability; MAV and ASM, indicating decreased muscle strength. This reduction of complexity can be interpreted as the central nervous system losing its ability to flexibly modulate motor unit recruitment and firing timing.

The observed variations in RMS between stroke and control groups reflect underlying motor unit recruitment abnormalities. Post-stroke weakness results from impaired recruitment of high-threshold motor units due to disrupted corticospinal input, leading to a reduction in RMS and signal energy during voluntary contractions. Conversely, the presence of hypertonic or spastic muscles may elevate RMS during rest or low-effort tasks, indicating increased background activity and reduced selective inhibition. These patterns are consistent with typical upper motor neuron lesions, in which decreased voluntary drive coexists with enhanced reflex excitability and compensatory activation of synergistic muscles.

MHW and MTW, demonstrating weaker energy distribution and amplitude variations; RMS, supporting diminished muscle activity magnitude and lower contraction levels [35]; and Sync, suggesting impaired muscle coordination [33]-all of which align with stroke-induced dysfunction (see Figure 4a–h). Furthermore, the neurological damage caused by stroke impairs the ability of MUs to activate and control muscle movements effectively [36]. This is reflected in the microscopic features, as stroke patients need to recruit more muscles on the affected side to perform the same gesture (see Figure 4i). Additionally, prolonged inactivity in the affected side of stroke patients can lead to a shift from fast-twitch fibers—responsible for explosive strength—to slow-twitch fibers, which are more suited for endurance, whereas less capable of generating strong contractions [37]. This change supports the observation that stroke patients exhibit lower AFR values on the affected side (see Figure 4j). A recent study suggests that with higher contraction levels and discharge rate, the signal-to-noise ratio tends to decrease [38]. This reduction in the signal-to-noise ratio complicates the ability of decomposition algorithms to identify and separate individual MU signals accurately. Moreover, higher contraction levels require the recruitment of more MUs to generate the necessary force. As more MUs fire simultaneously, their action potentials overlap more frequently, making it difficult to distinguish and separate individual motor unit action potentials during decomposition [39]. This further explains why fewer MUs were detected in healthy subjects than in stroke patients (see Figure 4i). Importantly, NMUs in this work represents the number of identifiable and retained motor units under our decomposition and quality-control criteria, rather than the true absolute motor unit count. Post-stroke neuromuscular control can exhibit altered recruitment and abnormal common synaptic input, while antagonist co-contraction is also frequently increased, which may increase mixture complexity. Under such pathological conditions, ICA-based decomposition may be challenged because its separation performance is linked to assumptions such as statistical independence, potentially leading to split or duplicate components and an inflated apparent NMUs. Therefore, the higher NMUs observed on the affected side is interpreted jointly with the applied quality-control procedures and should be viewed as an informative but decomposition-conditioned biomarker of post-stroke neuromuscular alterations.

Additionally, stroke patients often experience significant muscle atrophy, particularly in the affected limbs, due to impaired motor control and decreased ability to perform voluntary movements [40]. This reduced muscle use further diminishes their capacity to generate strong muscle contractions [41]. These discharge rate reductions could alter the precise match of motoneuron properties to the mechanical properties of innervated muscle fibers, reducing the efficiency of muscle contraction [42]. This further supports lower AFR values in stroke patients than in healthy subjects (see Figure 4j).

The classification results show that gestures like WE, TIMFP, and TIFP offer high accuracy (over 87%) for distinguishing Brunnstrom stages 4, 5, 6, and healthy controls, as shown in Figure 5. WE performed best with a 92.08% accuracy, as it involves large muscle groups (e.g., wrist extensors) that recover early in stroke rehabilitation. These muscles exhibit clear activity patterns that differentiate recovery stages and healthy controls. However, misclassifications occurred, particularly between adjacent Brunnstrom stages and between stage 6 and healthy controls. Stage 6, with nearly normal function, can still exhibit minor impairments, making it challenging to distinguish from healthy controls. Fine motor gestures like TIMFP and TIFP showed some misclassification as well, as these gestures involve smaller muscle groups and improve more gradually than gross motor movements. This variability in performance reflects the slower pace of recovery in fine motor skills and the difficulty in distinguishing subtle motor function differences in higher stages of recovery.

In regression analysis, gestures such as TIMFP, HC, and TIFP demonstrated strong correlations with stroke severity, reflected by high R-values (0.86, 0.74, 0.73) and low RMSEs (3.26, 4.42, 4.56). These fine motor gestures are highly sensitive to stroke severity, particularly in assessing hand and finger dexterity, which are often significantly impaired after a stroke. TIMFP and TIFP, involving thumb and index finger movements, are particularly effective for predicting fine motor control because these digits typically experience the most pronounced deficits in severe stages of stroke. HC, which involves hand closure, also strongly correlates with stroke severity, emphasizing the crucial role of hand function in grasping and object manipulation. Whereas various sensing modalities, such as kinematic and imaging-based methods [43], have been explored for predicting Fugl–Meyer scores, sEMG offers a complementary perspective by directly capturing neuromuscular activation rather than external movement outcomes or structural changes. This enables sEMG to detect subtle impairments in motor command and muscle coordination that may not produce visible kinematic deviations, particularly during the early stages of recovery when movement is limited. Therefore, sEMG-based assessment provides a unique advantage in quantifying residual voluntary control and fine motor recovery, which is well aligned with the correlations observed for fine motor gestures like TIMFP, TIFP, and HC. In contrast, gestures such as WE, WP, WF, and WS exhibited lower R-values and higher RMSEs, suggesting that they are less effective at identifying subtle variations in stroke severity. Overall, fine motor gestures are more informative for assessing detailed recovery of dexterity, whereas gross motor gestures are better suited for evaluating broader stages of motor restoration.

In this study, we explored the neurophysiological differences between stroke patients’ affected and unaffected sides, using HD-sEMG to extract macroscopic and microscopic features. These features were evaluated for their effectiveness in classifying motor recovery stages and predicting fine motor control restoration. Our findings highlight the importance of fine motor gestures for assessing recovery and also show that combining fine and gross motor gestures enhances stroke severity classification and motor recovery prediction.

However, there are some limitations to this study. First, the sample size, particularly for the healthy controls, was relatively small, which may limit the generalizability of the findings. Additionally, the data collected was limited to specific hand gestures, which may not fully represent the wide range of motor functions affected by stroke. In addition, recordings were obtained from forearm extensor/flexor regions; extending the framework to other muscle groups and functional tasks requires further validation. Residual artifacts (e.g., impedance variation and motion contamination) may still exist and will be systematically quantified in future work using dedicated quality-control procedures.

From a translational perspective, the proposed framework is suitable for longitudinal clinical monitoring and rehabilitation planning. In a practical workflow, HD-sEMG can be recorded at baseline and at follow-up visits using a small set of standardized gestures, and the extracted macroscopic/microscopic biomarkers together with the predicted Brunnstrom stages and UE-FMA score can be used as objective indicators of recovery status. Specifically, the outputs can support personalization in three concrete ways: (i) goal-oriented gesture selection, where therapists prioritize gestures that are most informative for the targeted clinical endpoint (e.g., gross recovery staging versus fine motor control), (ii) progress-driven dose adjustment, where changes in the predicted severity and biomarker profiles guide the progression of training intensity and task difficulty, and (iii) targeted correction of compensatory strategies, where longitudinal feature trends help identify abnormal activation patterns (e.g., excessive synergistic recruitment or inefficient activation) and prompt focused exercises to improve selective control. In future implementations, the same decision logic can be embedded into adaptive rehabilitation platforms or wearable systems to provide session-by-session quantitative feedback and enable closed-loop therapy in which training plans are updated according to the patient’s measured neuromuscular state.

## 6. Conclusions

In conclusion, this study demonstrates the effectiveness of HD-sEMG as a non-invasive tool for assessing muscle activity and monitoring rehabilitation progress in stroke patients. By analyzing both macroscopic and microscopic features, we identified significant differences in muscle coordination and strength between stroke patients and healthy controls. The high classification accuracy in distinguishing stroke stages and healthy controls, coupled with the strong regression model performance in predicting motor recovery, underscores the potential of HD-sEMG in evaluating stroke-induced motor impairment. These findings pave the way for integrating HD-sEMG into clinical practice, offering a valuable tool for personalized rehabilitation strategies and improving therapeutic outcomes for stroke patients.

## Data Availability

The original contributions presented in this study are included in the article. Further inquiries can be directed to the corresponding authors.

## Appendix A

### Subjects Information

**Table A1:** Personal information of the subjects.

Num	Gender	Stroke Duration (Years)	Affected Side	Etiology	Brunnstrom Stage	UE-FMA Scores
S1	M	4	Right	Ischemic	5	20
S2	M	7	Right	Ischemic	4	3
S3	M	17	Light	Ischemic	5	20
S4	M	2	Light	Ischemic	6	28
S5	F	1.5	Right	Ischemic	6	20
S6	M	3	Right	Ischemic	5	12
S7	M	40	Right	Hemorrhagic	4	4
S8	M	1.5	Right	Ischemic	5	9
S9	M	24	Light	Hemorrhagic	6	20
S10	M	17	Right	Ischemic	6	23
S11	M	5	Right	Ischemic	5	12
